# Rehabilitation in Adults with Complex Psychosis: A Clinician-Oriented Narrative Review of Multidimensional Approaches to Functional Recovery

**DOI:** 10.3390/medicina62050841

**Published:** 2026-04-28

**Authors:** Mario Pinzi, Andrea Fagiolini, Giacomo Gualtieri, Maria Beatrice Rescalli, Caterina Pierini, Alessia Santangelo, Benjamin Patrizio, Alessandro Cuomo

**Affiliations:** Department of Molecular Medicine, School of Medicine, University of Siena, 53100 Siena, Italy; mario.pinzi@student.unisi.it (M.P.); giacomo.gualtieri2@unisi.it (G.G.); m.rescalli@student.unisi.it (M.B.R.); c.pierini@student.unisi.it (C.P.); a.santangelo7@student.unisi.it (A.S.); benjamin.patrizio@student.unisi.it (B.P.); alessandro.cuomo@unisi.it (A.C.)

**Keywords:** complex psychosis, schizophrenia spectrum disorders, psychiatric rehabilitation, functional recovery, cognitive remediation, social cognition, metacognition, supported employment, integrated care, community-based rehabilitation

## Abstract

Complex psychosis is a clinically relevant rehabilitation construct rather than a formal diagnostic category and refers to psychotic illness associated with treatment-resistant symptoms, functional impairment, and additional cognitive, psychiatric, neurodevelopmental, or physical health complexity. In this clinician-oriented narrative review, we synthesised current evidence on rehabilitation interventions for adults with complex psychosis, integrating direct evidence from specialist rehabilitation settings with indirect evidence from schizophrenia-spectrum studies when clinically informative. We searched major clinical databases, prioritised guidelines, systematic reviews, meta-analyses, and controlled studies, and organised the synthesis by functional domain and pathway relevance. Evidence was strongest for cognitive remediation, particularly when combined with broader psychiatric rehabilitation or vocational support, for family interventions in relapse prevention, and for individual placement and support in competitive employment. Social–cognitive and metacognitive interventions appear clinically valuable, although transfer to real-world functioning is more variable. Community-based rehabilitation, supported accommodation, illness self-management, and ecological adaptation strategies remain central to functional recovery when embedded within multidisciplinary pathways. Digital and virtual interventions are promising adjuncts, but their efficacy remains heterogeneous and implementation challenges include engagement, privacy, and service integration. Overall, rehabilitation in complex psychosis is most convincing when it is personalised, measurement-based, and delivered through integrated service models linking assessment, intervention selection, supported living, and recovery-oriented care.

## 1. Introduction

Psychotic disorders, including schizophrenia and related spectrum conditions, represent some of the most severe and disabling psychiatric illnesses worldwide. Although advances in pharmacological treatments have significantly improved the management of positive symptoms such as hallucinations and delusions, many individuals continue to experience persistent functional impairments that affect social participation, independent living, and occupational functioning [1]. Over the past decades, growing attention has been directed toward the concept of functional recovery [2], reflecting the recognition that symptom remission alone does not adequately capture treatment outcomes in psychotic disorders [3]. A substantial body of research has demonstrated that improvements in clinical symptoms do not necessarily translate into better everyday functioning or quality of life. Cognitive deficits, negative symptoms, and social difficulties often persist even when psychotic symptoms are partially stabilized and represent major determinants of long-term disability [4,5]. Adults with complex psychosis present with a level of clinical and functional complexity that is not adequately captured by symptom severity alone. In line with NICE guidance, this construct refers to a primary psychotic disorder associated with treatment-resistant symptoms and functional impairment, usually accompanied by additional cognitive deficits, coexisting psychiatric conditions including substance misuse, neurodevelopmental conditions, and/or significant physical health comorbidity. These interacting difficulties substantially compromise social and everyday functioning and indicate the need for rehabilitation aimed at maximising independence rather than symptom control alone [6]. Within this evolving perspective, increasing attention has been directed toward the concept of complex psychosis, which is often used in clinical and service contexts to describe individuals with severe and enduring psychotic disorders characterized by persistent symptoms, cognitive impairment, negative symptoms, and substantial psychosocial disability requiring long-term and multidisciplinary care [7]. Although complex psychosis is not a formal diagnostic category within current classification systems, it represents a clinically meaningful construct that reflects the multidimensional needs of individuals with severe psychotic disorders. Research has consistently shown that functional outcomes in psychotic disorders are influenced by multiple interacting domains, including neurocognitive functioning, social cognition, metacognitive abilities, interpersonal skills, and vocational participation [8]. Impairments across these domains contribute significantly to difficulties in social integration and independent functioning, highlighting the limitations of treatment approaches that focus exclusively on symptom reduction [9]. In response to these challenges, rehabilitation interventions have increasingly been recognized as a central component of comprehensive care for individuals with psychotic disorders. A range of evidence-based approaches—including cognitive remediation, social cognition training, metacognitive interventions, psychosocial rehabilitation programs, and supported employment models—have been developed to target specific domains of functional impairment and promote recovery-oriented outcomes [10,11]. Importantly, effective rehabilitation requires not only the availability of evidence-based interventions but also systematic approaches to the assessment of functional needs, the development of individualized rehabilitation plans, and the evaluation of treatment outcomes. Multidisciplinary collaboration and the use of standardized functional assessment tools play a crucial role in ensuring that rehabilitation interventions are tailored to patients’ specific needs and recovery goals.

The aim of this clinician-oriented narrative review is not simply to summarise rehabilitation interventions, but to integrate them within a structured and clinically applicable framework for assessment, intervention selection, and pathway planning in adults with complex psychosis. In addition, this review seeks to identify key gaps in the current literature and to propose a pathway-based, multidimensional model of rehabilitation that emphasises the integration of cognitive, psychosocial, and service-level interventions.

## 2. Materials and Methods

This clinician-oriented narrative review was undertaken to synthesise rehabilitation interventions relevant to adults with complex psychosis and to translate the available evidence into practical implications for multidisciplinary care. The review was planned and reported in line with good-practice recommendations for narrative reviews and the SANRA framework, with additional attention to methodological transparency in relation to the search strategy, eligibility criteria, and narrative synthesis process [12].

### 2.1. Search Strategy

A structured literature search was conducted on 17 March 2026 in PubMed/MEDLINE. The search combined terms related to psychosis and schizophrenia-spectrum disorders with terms related to psychiatric rehabilitation, cognitive remediation, social cognition, metacognition, supported employment, supported accommodation, community rehabilitation, digital interventions, and functional recovery. The primary time window was restricted to the last 10 years (1 January 2016 to 17 March 2026), although earlier landmark studies were retained when necessary for conceptual or interventional context. In addition to database searches, relevant articles were identified through manual screening of reference lists of included studies and key review papers to ensure comprehensive coverage of clinically relevant interventions.

### 2.2. Eligibility Criteria

We included English-language studies involving adults aged 18 years or older with schizophrenia-spectrum or other primary psychotic disorders, provided that the publication addressed rehabilitation, psychosocial, cognitive, vocational, service-model, or technology-assisted interventions with clinically relevant functional implications. Eligible designs included practice guidelines, systematic reviews, meta-analyses, randomised controlled trials, controlled observational studies, and key implementation papers. We excluded paediatric studies, case reports, editorials, conference abstracts, protocols without results, and studies focused exclusively on biological outcomes without rehabilitation or functional relevance.

### 2.3. Study Selection and Data Extraction

Records were exported and de-duplicated prior to screening. Titles and abstracts were screened against the eligibility criteria, followed by full-text review of potentially relevant papers. For each included source, we extracted information on population, setting, intervention model, target functional domain, principal outcomes, and relevance to complex psychosis. Selection was guided by relevance to functional outcomes and clinical applicability to complex psychosis. When multiple sources were available for the same intervention, priority was given to recent systematic reviews, meta-analyses, and well-conducted randomized controlled trials, while individual studies were included when they provided clinically meaningful or illustrative data not captured in higher-level evidence.

### 2.4. Approach to Synthesis

Because of marked heterogeneity across populations, settings, interventions, and outcomes, a formal pooled quantitative synthesis was not attempted. Instead, the evidence was synthesised narratively and organised according to functional domains: neurocognition, social cognition, metacognition, social and community functioning, family and caregiver interventions, vocational rehabilitation, digital interventions, and integrated rehabilitation pathways. Within each section, greater interpretative weight was given to official guidelines, systematic reviews, meta-analyses, and controlled studies. The narrative synthesis explicitly considered differences in study design and level of evidence, distinguishing between meta-analyses, randomized controlled trials, controlled observational studies, and implementation research. Interpretations were weighted accordingly in each section. The search yielded over 1000 records from PubMed/MEDLINE; after screening, approximately 60 studies were included in the qualitative synthesis.

## 3. Defining Complex Psychosis

In this review, complex psychosis is understood as a rehabilitation-oriented clinical construct rather than a formal diagnostic category. Consistent with NICE guidance, it refers to a primary psychotic disorder associated with treatment-resistant or persistent symptoms and significant functional impairment, usually accompanied by one or more additional sources of complexity [6]. These may include cognitive deficits, coexisting psychiatric conditions such as substance misuse, neurodevelopmental disorders, and/or significant physical health comorbidity. What makes this population clinically distinctive is not merely symptom persistence, but the combined effect of these interacting difficulties on social participation, everyday functioning, and the individual’s ability to progress towards greater independence. This construct overlaps with, but should not be conflated with, treatment-resistant schizophrenia. Treatment-resistant schizophrenia is primarily a pharmacoresistance construct, typically defined by an inadequate response to at least two adequate antipsychotic trials [13]. By contrast, complex psychosis is broader and explicitly rehabilitation-focused: it encompasses functional disability, multi-domain complexity, and the need for sustained multidisciplinary care. For this reason, the concept is particularly useful when discussing care pathways, supported living, community rehabilitation, and the selection of interventions aimed at improving real-world functioning. Given the absence of formal diagnostic criteria, complex psychosis may be pragmatically operationalised using a combination of multidimensional clinical indicators (Table 1).

Given this pragmatic and dimensional definition, much of the available evidence derives from schizophrenia-spectrum populations and should be interpreted in terms of transferability to individuals with higher levels of clinical and functional complexity.

Accordingly, the evidence base should be read as a combination of direct rehabilitation evidence and indirect intervention evidence, with careful attention to transferability across settings and levels of complexity.

### 3.1. Evolution of Treatment Models in Psychosis

Historically, the treatment of psychotic disorders has been primarily oriented toward the reduction in psychotic symptoms through pharmacological interventions. The introduction of antipsychotic medications in the mid-twentieth century represented a major turning point in the management of schizophrenia and related disorders, significantly improving the control of positive symptoms such as hallucinations and delusions. However, longitudinal research progressively demonstrated that symptom remission alone does not necessarily translate into functional recovery. Many individuals with schizophrenia continue to experience substantial impairments in social functioning, independent living, and occupational participation despite partial symptom stabilization [1]. These findings contributed to a shift in the conceptualization of treatment outcomes in psychotic disorders. Increasing evidence indicated that cognitive deficits and functional impairment represent central determinants of long-term disability, often independently from the severity of psychotic symptoms [4,5]. In parallel, the recovery movement emerged as an influential framework in mental health care. This perspective emphasized the importance of personal recovery, empowerment, and social inclusion as key goals of treatment, moving beyond the traditional focus on symptom reduction. Recovery-oriented models highlight the role of meaningful life goals, autonomy, and participation in community life in shaping long-term outcomes for individuals with severe mental illness [14,15]. Within this evolving conceptual framework, increasing attention has been directed toward the development of rehabilitation interventions targeting cognitive, social, and vocational functioning, reflecting the recognition that comprehensive care for psychotic disorders requires addressing multiple domains of impairment.

### 3.2. Domains of Functional Impairment

**Figure 1:**
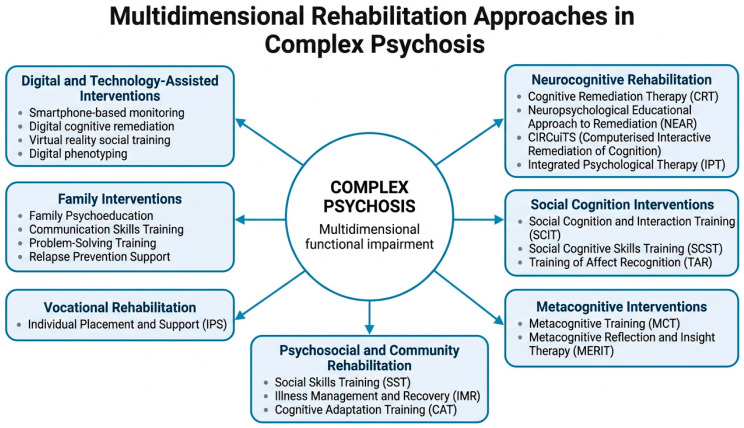
Conceptual model of functional impairment and rehabilitation targets in complex psychosis. Persistent psychotic symptoms, cognitive dysfunction, social–cognitive impairment, metacognitive difficulties, and contextual barriers interact to affect independent living, social participation, and vocational functioning. Rehabilitation interventions are mapped onto these domains within a recovery-oriented framework.

A central feature of complex psychosis is the presence of impairments across multiple functional domains that contribute to long-term disability. Research has consistently demonstrated that functional outcomes in schizophrenia are influenced by a range of interacting factors, including neurocognitive deficits, social cognitive impairments, negative symptoms, and environmental barriers [16]. Neurocognitive deficits represent one of the most extensively studied domains in schizophrenia. Impairments in attention, working memory, processing speed, and executive functioning are widely documented and strongly associated with functional disability [17]. In addition to basic neurocognitive functions, social cognition plays a crucial role in determining social functioning. Social cognitive processes include the ability to recognize emotions, interpret social cues, and understand the mental states of others. Deficits in these abilities are associated with difficulties in interpersonal relationships and community participation [9]. Another important dimension concerns metacognition, which refers to the capacity to reflect upon and integrate one’s own thoughts and experiences. Impairments in metacognitive processes have been linked to difficulties in insight, identity formation, and engagement in recovery processes [18]. Finally, functional outcomes in psychotic disorders are strongly influenced by social and vocational participation, including the ability to maintain employment, independent living, and meaningful social relationships. These domains represent key indicators of recovery and are increasingly recognized as central targets of psychiatric rehabilitation interventions. Taken together, these multidimensional impairments highlight the need for integrated rehabilitation approaches capable of addressing cognitive, social, and functional domains simultaneously in individuals with complex psychosis. The multidimensional structure of functional impairment and its relationship with rehabilitation targets is summarised in Figure 1.

## 4. Rehabilitation Interventions in Complex Psychosis

Given the multidimensional nature of disability in complex psychosis, rehabilitation interventions are often conceptualized according to the functional domains they aim to target. Cognitive, social, and vocational impairments interact in shaping real-world functioning, and rehabilitation programs have therefore been developed to address these domains through structured and evidence-based interventions. Among these approaches, cognitive remediation, social cognitive training, metacognitive interventions, psychosocial rehabilitation programs, and vocational rehabilitation have received increasing attention in both research and clinical practice.

### 4.1. Neurocognitive Rehabilitation

Neurocognitive deficits represent one of the most consistent and disabling features of schizophrenia and related psychotic disorders. Impairments in attention, working memory, processing speed, and executive functioning are widely documented and are strongly associated with poor functional outcomes in areas such as social functioning, independent living, and employment [4,17]. These impairments tend to remain relatively stable throughout the course of illness and are only minimally responsive to pharmacological treatments, highlighting the need for targeted rehabilitation approaches. Cognitive remediation therapy (CRT) refers to a set of behavioral training interventions designed to improve cognitive processes through repeated practice and the development of compensatory strategies. The theoretical basis of CRT lies in principles of neuroplasticity and learning theory, suggesting that repeated cognitive exercises can strengthen neural networks and improve cognitive functioning. In addition, many cognitive remediation programs emphasize the importance of strategy learning and generalization, aiming to facilitate the transfer of cognitive improvements to everyday functioning [10]. Among the different cognitive remediation approaches, the Neuropsychological Educational Approach to Remediation (NEAR) is one of the most widely implemented models. NEAR combines computer-based cognitive exercises with educational principles and motivational strategies designed to enhance engagement and promote the generalization of cognitive gains to real-life goals. The intervention typically includes structured sessions in which patients practice cognitive tasks targeting attention, memory, and executive functioning while therapists provide guidance on cognitive strategies and encourage reflection on how these skills can be applied in everyday activities [19]. Another important cognitive remediation model is CIRCuiTS (Computerised Interactive Remediation of Cognition–Training for Schizophrenia), which places a stronger emphasis on metacognitive awareness and strategy use. The program includes interactive computerized tasks aimed at improving cognitive performance, combined with therapist-led discussions designed to increase patients’ awareness of their cognitive processes and to facilitate the transfer of cognitive strategies to real-world situations. Randomized controlled trials have demonstrated that CIRCuiTS can lead to improvements in cognitive functioning and may support broader rehabilitation outcomes [10]. An earlier but still influential approach is Integrated Psychological Therapy (IPT), which combines neurocognitive training with modules targeting social perception, communication skills, and problem-solving. IPT is typically delivered in a group format and follows a hierarchical structure in which cognitive exercises are progressively linked to social and behavioral skills. This multimodal structure reflects the assumption that improvements in basic cognitive processes can facilitate higher-order social and functional abilities [20]. Overall, a substantial body of research supports the effectiveness of cognitive remediation interventions in schizophrenia. Meta-analyses have consistently demonstrated moderate improvements in cognitive performance across multiple domains, with additional benefits for functional outcomes when cognitive remediation is combined with psychosocial rehabilitation or vocational interventions [10,21]. These findings suggest that neurocognitive rehabilitation represents a key component of treatment for individuals with complex psychosis, particularly when integrated within broader rehabilitation programs aimed at improving social functioning and community participation [22].

### 4.2. Social Cognitive Interventions

Social cognition refers to the set of cognitive processes involved in perceiving, interpreting, and responding to social information. These processes include abilities such as emotion recognition, theory of mind, attributional style, and social perception. A growing body of research has shown that impairments in social cognition are highly prevalent in schizophrenia and represent a key determinant of functional outcomes, particularly in domains such as interpersonal relationships and community participation [4,9]. Importantly, social cognition appears to mediate the relationship between basic neurocognitive functioning and real-world social outcomes. Individuals with schizophrenia often show difficulties in recognizing facial emotions, understanding others’ intentions, and interpreting social cues, which may contribute to social withdrawal and impaired interpersonal functioning [9]. In response to these findings, several structured rehabilitation interventions have been developed specifically to target social cognitive processes. One of the most widely studied programs is Social Cognition and Interaction Training (SCIT). SCIT is a structured group-based intervention designed to improve emotion perception, theory of mind, and attributional style. The program typically consists of multiple modules delivered over several sessions and includes psychoeducation about social cognition, exercises aimed at improving emotion recognition, and activities designed to reduce attributional biases such as the tendency to misinterpret ambiguous social situations as threatening. Role-playing and group discussions are commonly used to help participants practice social interpretation and develop more adaptive responses to social situations [9]. Another important intervention is Social Cognitive Skills Training (SCST), which focuses on enhancing specific components of social cognition, including emotion recognition and theory of mind. SCST is typically delivered through structured sessions that include computerized exercises, video-based training, and therapist-guided feedback. Participants practice identifying emotional expressions and interpreting social situations, with the aim of improving their ability to navigate interpersonal interactions in everyday contexts [23]. A further approach is Training of Affect Recognition (TAR), which focuses specifically on improving the ability to recognize facial emotional expressions. TAR typically involves repeated exposure to facial stimuli representing different emotional states, combined with feedback and strategy training. This approach is based on the assumption that improved emotion recognition can enhance interpersonal understanding and social functioning [24]. Evidence supporting social cognitive interventions has been summarized in several meta-analyses. A meta-analysis by Kurtz and Richardson demonstrated that social cognitive training programs produce significant improvements in emotion recognition and theory of mind in individuals with schizophrenia [25]. Importantly, improvements in social cognition may translate into better interpersonal functioning and greater social participation, particularly when social cognitive training is integrated with broader psychosocial rehabilitation programs. Taken together, these findings suggest that social cognitive interventions represent an important component of rehabilitation for individuals with complex psychosis, as they target cognitive processes directly involved in everyday social interactions and community functioning. Social–cognitive interventions show consistent benefits for proximal targets such as emotion recognition and theory of mind, but their effects on broader social functioning appear more variable and may depend on treatment intensity, intervention design, and opportunities for real-world practice. In complex psychosis, these approaches are best conceptualised as targeted components of a broader rehabilitation plan rather than stand-alone solutions. Evidence from randomized controlled trials and meta-analyses supports improvements in proximal social cognitive outcomes; however, effects on broader real-world functioning are less consistent, and findings remain heterogeneous across study designs and intervention formats [25].

### 4.3. Metacognitive Interventions

Metacognition refers to the ability to reflect upon and integrate one’s own thoughts, emotions, and experiences, as well as to understand the mental states of others. In psychotic disorders, impairments in metacognitive processes are associated with reduced insight, difficulties in identity integration, impaired social functioning, and limited engagement in recovery-oriented processes [18]. Individuals with schizophrenia often show deficits in recognizing cognitive biases, reflecting on their own beliefs, and integrating complex psychological experiences into coherent narratives, which may contribute to the persistence of delusional beliefs and difficulties in adapting to social and environmental demands [26]. In response to these deficits, structured psychological interventions targeting metacognitive processes have been developed. Among these, Metacognitive Training (MCT) is one of the most widely disseminated approaches. MCT is typically delivered in a group format and integrates elements of cognitive–behavioral therapy, cognitive remediation, and psychoeducation. It specifically targets cognitive biases commonly observed in psychosis—such as jumping to conclusions, attributional biases, and overconfidence in erroneous beliefs—through a series of structured sessions combining psychoeducation, experiential exercises, and guided group discussions [26,27]. The goal is to foster awareness of biased thinking patterns and promote more flexible and reflective reasoning styles. Evidence suggests that MCT is associated with small-to-moderate improvements in positive symptoms, particularly in reducing delusional conviction, as well as decreases in cognitive biases. However, effects on broader functional outcomes and long-term recovery remain less consistent across studies [27]. Another relevant intervention is Metacognitive Reflection and Insight Therapy (MERIT), an individual psychotherapy approach that focuses on enhancing patients’ capacity for self-reflection and integration of personal experiences into coherent narratives. Unlike MCT, which directly targets specific cognitive biases, MERIT aims to improve global metacognitive capacity through therapeutic dialogue and exploration of subjective experience, with the goal of supporting insight, emotional regulation, and recovery processes [18,28]. Compared with MCT, the evidence base for MERIT is more limited and heterogeneous, consisting primarily of smaller clinical trials and observational studies. Overall, when compared to more established interventions such as cognitive remediation or supported employment, the empirical support for metacognitive interventions remains less robust, with fewer large randomized controlled trials and greater variability in study designs. Therefore, these approaches should be considered promising, theory-driven interventions that may complement broader rehabilitation strategies, particularly within integrated and personalized treatment models.

### 4.4. Social and Community Rehabilitation

Impairments in social functioning and community participation are among the most disabling aspects of complex psychosis. Even when positive symptoms are partially controlled, many individuals continue to experience difficulties in interpersonal communication, problem-solving, illness self-management, and independent living. These limitations often contribute to long-term social exclusion, poor quality of life, and repeated service use. For this reason, psychosocial and community-based rehabilitation interventions have been developed to improve everyday functioning in ecologically relevant contexts. One of the most established approaches in this area is Social Skills Training (SST). SST is grounded in social learning theory and behavioral principles, and is based on the assumption that interpersonal skills can be taught, practiced, and reinforced through structured training. The model typically includes therapist modeling, role-playing, behavioral rehearsal, corrective feedback, and homework practice. Sessions usually focus on concrete domains such as conversational skills, assertiveness, conflict management, medication communication, and problem-solving in daily life. The goal is not only to improve performance in structured settings, but also to facilitate the generalization of these skills to real interpersonal situations. Meta-analytic evidence has shown that SST is associated with significant benefits in social skills and interpersonal functioning in schizophrenia [29]. Another important model is Illness Management and Recovery (IMR). IMR is based on the integration of psychoeducation, relapse prevention, motivational strategies, and recovery-oriented principles. The theoretical rationale is that better illness knowledge, early recognition of warning signs, adherence to treatment, and coping skills can improve long-term stability and support personal recovery goals. In practice, IMR is usually delivered through individual or group sessions and includes modules on psychoeducation, stress-vulnerability models, relapse prevention, coping with persistent symptoms, medication management, and goal setting. Unlike more narrowly symptom-focused approaches, IMR explicitly incorporates the patient’s personal recovery goals and aims to strengthen self-management and active engagement in care. Mueser and colleagues reviewed the research supporting this model and concluded that illness management interventions can improve illness knowledge, treatment participation, and relapse prevention capacities in people with severe mental illness [30]. A further highly relevant approach for complex psychosis is Cognitive Adaptation Training (CAT). CAT is based on the premise that many everyday functional difficulties in schizophrenia derive not only from symptoms, but also from persistent executive and attentional impairments that interfere with initiation, organization, and follow-through in daily activities. Rather than attempting to directly normalize cognition, CAT uses environmental supports and compensatory strategies to bypass cognitive deficits. The intervention is usually delivered in the person’s home or real-life environment and includes the use of signs, checklists, alarms, pill containers, structured object placement, and routines tailored to the patient’s cognitive profile and functional needs. In this sense, CAT is highly ecological and especially suitable for individuals with marked disability in everyday functioning. Early randomized controlled evidence showed that CAT improved adaptive functioning in outpatients with schizophrenia compared with standard treatment conditions, and later work suggested sustained benefits when CAT was implemented in community-oriented settings [31,32]. Taken together, these interventions address complementary aspects of functional disability in complex psychosis. SST primarily targets interpersonal competence and social behavior, IMR focuses on illness self-management and recovery-oriented goal setting, and CAT aims to improve adaptive functioning through ecological supports in everyday environments. Their shared clinical value lies in moving beyond symptom reduction and directly addressing the skills and supports required for community participation and independent living. Social and community rehabilitation should be positioned as pathway-based care rather than a collection of isolated techniques. Skills training, illness self-management, cognitive adaptation, supported accommodation, and community rehabilitation work best when they are linked to personalised goals, ecological practice, and graded progression towards greater independence.

Evidence supporting these interventions derives from a combination of randomized controlled trials and observational studies, with stronger support for structured skills-based approaches, while ecological and community-based interventions are more frequently informed by real-world and implementation research [29,30,31,32].

### 4.5. Family and Caregiver Involvement in Psychosocial Rehabilitation

Family involvement should be presented as an evidence-based relapse prevention strategy rather than solely as a contextual component of care. Contemporary evidence indicates that several family intervention models reduce relapse risk, with family psychoeducation showing particularly consistent effects, although acceptability and implementation vary across services. Family work is therefore best understood as a structured component of rehabilitation that can improve treatment engagement, reduce interpersonal stress, and support more stable community living [33,34,35]. Structured family interventions typically include several core components. First, psychoeducation provides relatives with information about the nature of psychotic disorders, symptom trajectories, treatment options, and early warning signs of relapse. This component aims to improve illness understanding and reduce misconceptions or stigma related to the disorder [36]. Second, many programs incorporate communication skills training, helping family members develop more constructive interaction styles and reduce critical or hostile communication patterns that may increase stress for the patient. Third, problem-solving training is often used to support families in managing everyday difficulties related to symptoms, social functioning, or treatment adherence. These approaches typically involve structured sessions in which clinicians guide families in identifying problems, generating solutions, and evaluating outcomes. Evidence from systematic reviews and meta-analyses suggests that family interventions may contribute to reduced relapse rates, fewer hospitalizations, and improvements in treatment adherence and caregiver burden among individuals with psychotic disorders [37,38]. Despite the documented benefits, the implementation of family interventions in routine mental health services remains inconsistent. Barriers may include limited availability of trained clinicians, organizational constraints, and challenges in engaging family members who may experience significant caregiving burden or stigma associated with the disorder [39]. Overall, integrating family-based approaches within psychosocial rehabilitation programs may represent an important strategy for promoting more supportive social environments, improving treatment engagement, and enhancing functional recovery in individuals living with psychotic disorders.

Among psychosocial interventions, family-based approaches are supported by some of the most consistent evidence, including multiple meta-analyses and randomized studies demonstrating reductions in relapse and hospitalization [37,38].

### 4.6. Vocational Rehabilitation

Vocational functioning represents a central dimension of recovery in psychotic disorders. Employment is associated not only with financial independence, but also with social inclusion, personal identity, daily structure, and self-efficacy. However, individuals with schizophrenia and related psychotic disorders frequently face major barriers to employment, including cognitive deficits, negative symptoms, stigma, fragmented work histories, and limited access to specialized vocational services [40,41]. In complex psychosis, these barriers are often compounded by long illness duration, repeated hospitalizations, and marked psychosocial disability, making vocational rehabilitation a particularly important component of treatment. The best-supported model of vocational rehabilitation in severe mental illness is Individual Placement and Support (IPS), the evidence-based form of supported employment. IPS is based on a recovery-oriented and person-centered approach, and departs from traditional vocational rehabilitation models that require lengthy pre-vocational training before entering the workforce. Instead, IPS is built on the principle of rapid placement in competitive employment, followed by ongoing individualized support. Its core assumption is that most people with severe mental illness who wish to work can do so successfully if they receive timely assistance, integration with mental health care, and support tailored to their preferences and needs [42]. The IPS model is defined by a set of well-established principles. These include a focus on competitive employment rather than sheltered work, eligibility based on the individual’s choice to work rather than “work readiness,” rapid job search, attention to patient preferences, integration of vocational specialists within mental health teams, individualized job support, and counseling on benefits and work-related financial issues. In practical terms, IPS usually involves close collaboration between mental health professionals and employment specialists, active job development in the community, and ongoing support once employment has been obtained. This makes IPS particularly suited to individuals with complex psychosis, for whom vocational goals often need to be pursued alongside ongoing psychiatric and psychosocial care. A large body of evidence has demonstrated the superiority of IPS over traditional vocational approaches. One of the most influential studies was the multicenter European randomized controlled trial by Burns and colleagues, which compared IPS with standard vocational services across several countries. The study found that individuals receiving IPS were significantly more likely to obtain competitive employment and worked more hours than those receiving conventional services [43]. This trial was particularly important because it demonstrated that IPS could be successfully implemented beyond the United States and within different mental health service systems. Subsequent reviews and meta-analyses have consistently confirmed these findings. Bond and colleagues showed that IPS is associated with higher rates of competitive employment compared with traditional vocational rehabilitation and that its benefits are generalizable across a range of countries and service settings [11]. Later work by Drake and colleagues further reinforced the conclusion that supported employment, and IPS in particular, remains the strongest evidence-based vocational intervention for people with severe mental illness [42]. The evidence for IPS is among the most robust in psychiatric rehabilitation, supported by multiple randomized controlled trials and meta-analyses across different countries and service settings. In addition to increasing employment rates, IPS may also have broader clinical and functional effects. Participation in competitive work can improve self-esteem, social role functioning, and perceived quality of life, while also supporting recovery-oriented outcomes such as autonomy and community participation. Nevertheless, job retention remains a challenge for some individuals, especially those with persistent cognitive deficits or severe negative symptoms. For this reason, recent research has explored the integration of IPS with other rehabilitation approaches, particularly cognitive remediation, in order to improve both job acquisition and maintenance in patients with more complex functional profiles [21]. Vocational rehabilitation should be framed as a core recovery outcome rather than a secondary adjunct. Individual Placement and Support remains the intervention with the clearest evidence for competitive employment, and its effects may be strengthened when combined with cognitive remediation for people whose cognitive difficulties limit job acquisition or retention.

### 4.7. Digital and Technology-Assisted Rehabilitation in Psychosis

Digital and technology-assisted interventions are increasingly being investigated as adjunctive tools within psychosis rehabilitation [44]. Their primary proposed functions include symptom and relapse monitoring, support for self-management, delivery of cognitive or psychosocial exercises, and the use of immersive environments to practice skills in ecologically valid settings [45,46].

However, the current evidence base remains heterogeneous in scope, quality, and clinical maturity, with many tools lacking robust scientific validation. Psychosis-specific digital trials suggest that these approaches are feasible and generally acceptable to service users, but efficacy findings remain mixed [44,47].

For example, the Actissist randomised clinical trial demonstrated that a CBT-informed application was safe and engaged users, yet it was not superior to an active symptom-monitoring control regarding primary psychotic outcomes [48]. By contrast, the EMPOWER feasibility cluster trial suggested that blended digital monitoring—combining peer support and clinical triage—is acceptable and justifies definitive trials for relapse prevention, though current results are not yet confirmatory of overall effectiveness [49].

Virtual reality-based interventions also show promise for social, cognitive, and vocational practice in controlled yet realistic environments, although evidence is still considered preliminary [50]. Furthermore, digital phenotyping via wearables and smartphones offers objective longitudinal data on sleep and mobility that are often inaccessible through standard clinical interviews [51].

Taken together, digital interventions should presently be viewed as adjunctive components of rehabilitation rather than replacements for structured multidisciplinary care. Their clinical value depends significantly on sustained engagement, human supervision to facilitate the transfer of skills, robust privacy safeguards, and seamless integration within established rehabilitation pathways [52].

### 4.8. Integrated, Multimodal and Outcome-Oriented Rehabilitation

Integrated rehabilitation for individuals with complex psychosis is best understood as a coordinated and pathway-based process rather than a mere collection of isolated interventions. This approach is fundamental because the multidimensional nature of the disorder—where persistent symptoms, neurocognitive deficits, and social–cognitive dysfunctions interact—frequently limits the effectiveness of treatments when they are delivered in a fragmented manner. Contemporary models therefore emphasize a seamless continuity of care across inpatient, community, and supported living environments, ensuring that all efforts are organized around the patient’s personalized recovery goals rather than symptom reduction alone [53].

The clinical utility of this integrated framework is largely rooted in the synergy achieved by combining different evidence-based treatments. For instance, meta-analytic research has shown that the impact of cognitive remediation on psychosocial functioning is significantly amplified when it is provided alongside adjunctive psychiatric rehabilitation, suggesting that improved cognition allows patients to benefit more from other rehabilitative activities [21]. Similarly, the integration of cognitive remediation with vocational programs like Individual Placement and Support (IPS) has proven highly effective in fostering competitive employment by helping patients overcome cognitive barriers to work [10]. Recent studies in real-world settings have further demonstrated the feasibility and effectiveness of protocols combining stable pharmacological treatment with computer-assisted cognitive remediation and social skills training to improve global functioning [54].

A central component of this rehabilitative model is the implementation of a comprehensive, multidisciplinary assessment that serves as the foundation for a functional formulation. Standardized tools such as the MATRICS Consensus Cognitive Battery (MCCB) are recommended to establish a baseline profile and monitor progress over time [55]. By integrating psychiatric, psychological, and social perspectives, the multidisciplinary team can tailor the selection and sequencing of interventions to meet the specific needs of the individual, ensuring that treatment gains are translated into meaningful community participation.

Finally, this integrated approach is inherently outcome-oriented and grounded in a recovery-focused culture. Systematic evaluation using objective measures like the Personal and Social Performance Scale (PSP) allows for the longitudinal tracking of social and personal functioning [56].

At the same time, the inclusion of patient-reported outcomes ensures that the focus remains on subjective recovery, hope, and the achievement of self-directed goals. Models such as the Active Recovery Triad (ART) underscore the vital importance of active collaboration between service users, families, and professionals to build strong support systems that facilitate long-term integration and a meaningful life [53].

**Figure 2:**
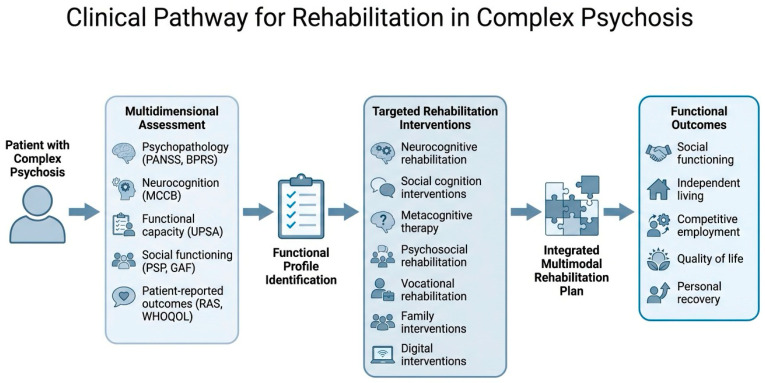
Assessment-to-intervention pathway for adults with complex psychosis. Multidimensional assessment informs personalised selection of cognitive, psychosocial, family, vocational, and digital adjunctive interventions, embedded within multidisciplinary rehabilitation care.

A structured assessment-to-intervention pathway reflecting this integrated model is illustrated in Figure 2.

To facilitate clinical translation of the reviewed evidence, Table 2 summarises the main assessment-to-intervention links across key functional domains relevant to complex psychosis rehabilitation.

## 5. Discussion and Clinical Implications

The main contribution of this review is to reposition rehabilitation in complex psychosis as a clinically structured, pathway-based process rather than a loose aggregation of adjunctive techniques. The available evidence does not support a one-size-fits-all model; instead, it consistently favours a formulation-driven approach in which intervention selection follows a multidimensional assessment of cognition, social cognition, metacognition, functional capacity, and vocational goals [57,58]. Importantly, the strength of evidence is uneven across domains and study designs, with meta-analyses and randomized controlled trials supporting some interventions (e.g., cognitive remediation and supported employment), while other areas rely more heavily on smaller, heterogeneous, or observational studies. Cognitive remediation shows robust and replicated effects on cognitive performance, with additional benefits for functional outcomes when embedded within broader rehabilitation programmes [10,22]. Family interventions represent one of the most reliable relapse prevention strategies, with recent meta-analytic evidence confirming reductions in relapse and rehospitalisation [37,59]. Vocational rehabilitation, particularly Individual Placement and Support, remains the gold-standard intervention for improving competitive employment outcomes, with consistent effectiveness across international trials and service contexts [60,61]. By contrast, the evidence base for digital interventions and some metacognitive approaches remains less mature. While these interventions are theoretically grounded and clinically promising, current findings are heterogeneous and insufficient to support routine implementation as stand-alone treatments [26,44]. Their role is therefore best conceptualised as adjunctive, particularly when integrated within structured rehabilitation pathways.

A further complexity lies in the indirect nature of much of the available evidence. Several gaps in the current literature emerge from this synthesis. First, relatively few studies explicitly focus on populations meeting criteria for complex psychosis, limiting the precision of clinical generalisation. Second, while many interventions demonstrate efficacy under controlled conditions, there is limited evidence regarding their implementation, scalability, and long-term sustainability in real-world rehabilitation pathways. Third, the interaction between different rehabilitation domains (e.g., cognitive, social, and vocational) remains insufficiently explored, despite its clear clinical relevance. A substantial proportion of studies are conducted in schizophrenia-spectrum populations without explicitly operationalising complex psychosis, limiting the precision of generalisation. This reflects a broader limitation in the field, where functional complexity is clinically recognised but inconsistently defined in research settings. From a service perspective, outcomes appear to depend as much on pathway organisation as on the specific interventions delivered. Clinical guidelines and contemporary rehabilitation literature converge in emphasising the importance of integrated care pathways, supported accommodation, community rehabilitation teams, and systematic attention to physical health and social inclusion [6,62]. Within this framework, the key clinical question shifts from whether a single intervention is effective in isolation to how evidence-based components can be appropriately sequenced and embedded within a coherent multidisciplinary system that facilitates functional recovery and progression towards independence. Cognitive, social, and vocational interventions target partially overlapping mechanisms; however, their clinical impact appears to depend less on their standalone efficacy than on their integration within coordinated care pathways. Emerging evidence suggests that the effectiveness of rehabilitation is mediated by system-level organisation and continuity of care rather than by isolated intervention effects [53]. This supports a broader conceptual shift from intervention-centred models to system-oriented approaches to rehabilitation. Implementation remains a critical challenge. Despite strong evidence for several rehabilitation interventions under controlled conditions, their availability, fidelity, and scalability in routine practice remain inconsistent. Organisational constraints, workforce limitations, and unequal access to specialised rehabilitation services represent persistent barriers to translation [63]. Addressing these gaps requires not only further efficacy research, but also a stronger focus on implementation science, service design, and workforce development. Ultimately, future progress in the field will depend not only on refining individual interventions, but on developing scalable, system-level models capable of delivering personalised, integrated rehabilitation across diverse clinical settings.

## 6. Limitations of the Review

This review has limitations. First, as a narrative review, it does not provide the exhaustive capture or formal risk-of-bias procedures expected of a full systematic review. Secondly, the literature summarised here is heterogeneous across populations, settings, intervention formats, and outcome measures. Thirdly, a considerable proportion of the evidence comes from schizophrenia-spectrum studies rather than cohorts explicitly defined according to contemporary complex psychosis criteria, which limits direct inferential strength. Fourthly, service-model and implementation evidence remains less developed than intervention-specific evidence. These limitations notwithstanding, a transparent narrative approach remains useful for integrating clinical and service-level evidence across domains that are rarely synthesised together in rehabilitation practice.

## 7. Future Directions

Future research should prioritise trials and service evaluations in populations explicitly characterised according to contemporary complex psychosis criteria, rather than relying solely on broader schizophrenia-spectrum samples. In addition, there is a need for studies explicitly designed to test integrated, pathway-based rehabilitation models, rather than isolated interventions, in order to better reflect real-world clinical practice. Hybrid effectiveness–implementation studies are especially needed to examine how domain-targeted interventions perform when embedded in real rehabilitation pathways, including supported accommodation and community rehabilitation services. Research should also move beyond symptom endpoints and routinely include everyday functioning, occupational outcomes, patient-reported recovery, physical health, and transition markers such as movement to less supported settings. Finally, digital and virtual interventions should be evaluated as adjuncts to multidisciplinary care, with particular attention to sustained engagement, privacy, and equity of access.

## 8. Conclusions

Rehabilitation in complex psychosis should be understood as a central component of care rather than as a late or optional add-on. The available evidence supports a personalised, multidisciplinary, and pathway-based model in which cognitive, psychosocial, family, vocational, and community interventions are selected according to clearly defined functional needs and recovery goals. The most defensible interpretation of the literature is that functional recovery is maximised not by any single technique, but by the coherent integration of evidence-based components within specialist rehabilitation care. A clinician-oriented approach that explicitly distinguishes direct from indirect evidence may help translate this literature into more precise and implementable practice.

## Figures and Tables

**Table 1:** Pragmatic clinical indicators of complex psychosis.

Domain	Clinical Indicator
Symptoms	Persistent or treatment-resistant psychotic symptoms (e.g., inadequate response to ≥2 antipsychotic trials)
Functioning	Marked functional impairment in independent living, social, or occupational domains
Cognition	Significant neurocognitive deficits affecting everyday functioning
Comorbidity	Co-occurring psychiatric or neurodevelopmental conditions (e.g., substance use, personality disorders, autism spectrum traits)
Physical Health	Physical comorbidity contributing to disability
Social Context	Social disadvantage or environmental instability (e.g., supported housing needs, unemployment, limited network)
Service Use	High service use (e.g., repeated hospitalisations, need for long-term multidisciplinary care)

Note: Not all indicators need to be present simultaneously; complex psychosis reflects the cumulative burden of multidimensional difficulties requiring integrated rehabilitation approaches.

**Table 2:** Main assessment-to-intervention associations for clinician-oriented rehabilitation planning in complex psychosis.

Functional Problem/Target	Suggested Assessment Domain	Example Tools	Rehabilitation Option(s)
Neurocognitive deficits	Neurocognition	MCCB	Cognitive remediation (NEAR, CIRCuiTS), CAT
Social cognition impairment	Social cognition	FEIT, Hinting Task	SCIT, SCST
Poor social functioning	Social functioning	PSP, SFS	Social Skills Training, community rehabilitation
Impaired daily living skills	Functional capacity	UPSA	Cognitive Adaptation Training, supported living
High relapse risk/family stress	Family/relapse	Relapse history	Family psychoeducation
Unemployment/vocational impairment	Vocational functioning	Work history	Individual Placement and Support (IPS)

## Data Availability

No new data were created or analyzed in this study.
